# Evolution of Interferons and Interferon Receptors

**DOI:** 10.3389/fimmu.2017.00209

**Published:** 2017-03-02

**Authors:** Chris J. Secombes, Jun Zou

**Affiliations:** ^1^Scottish Fish Immunology Research Centre, University of Aberdeen, Aberdeen, UK

**Keywords:** interferon, interferon receptor, evolution, retrotransposition, gene duplication, fish, vertebrate

## Abstract

The earliest jawed vertebrates (Gnathostomes) would likely have had interferon (IFN) genes, since they are present in extant cartilaginous fish (sharks and rays) and bony fish (lobe-finned and ray-finned fish, the latter consisting of the chondrostei, holostei, and teleostei), as well as in tetrapods. They are thought to have evolved from a class II helical cytokine ancestor, along with the interleukin (IL)-10 cytokine family. The two rounds of whole genome duplication (WGD) that occurred between invertebrates and vertebrates (1) may have given rise to additional loci, initially containing an IL-10 ancestor and IFN ancestor, which have duplicated further to give rise to the two loci containing the IL-10 family genes, and potentially the IFN type I and IFN type III loci (2). The timing of the divergence of the IFN type II gene from the IL-10 family genes is not clear but was also an early event in vertebrate evolution. Further WGD events at the base of the teleost fish, and in particular teleost lineages (cyprinids, salmonids), have duplicated the loci further, giving rise to additional IFN genes, with tandem gene duplication within a locus a common occurrence. Finally, retrotransposition events have occurred in different vertebrate lineages giving rise to further IFN loci, with large expansions of genes at these loci in some cases. This review will initially explore the likely IFN system present in the earliest Gnathostomes by comparison of the known cartilaginous fish genes with those present in mammals and will then explore the changes that have occurred in gene number/diversification, gene organization, and the encoded proteins during vertebrate evolution.

## The Interferon (IFN) Pathway in Early Gnathostomes

With the recent sequencing of the elephant shark genome (3) it has become apparent that while some differences exist in the antiviral pathways present in cartilaginous fish and mammals, a fully functional IFN system is present in these early vertebrates, as already well established in bony fish (Osteichthyes) (4–7). As outlined below, this includes pattern recognition receptors (PRRs) to detect virus, PRR signaling molecules to effect IFN induction, the IFN genes themselves, their receptors, and associated signaling molecules to trigger antiviral responses, and the IFN-stimulated genes (ISGs) that act to inhibit viral replication in the host.

### Sensors

**Figure 1:**
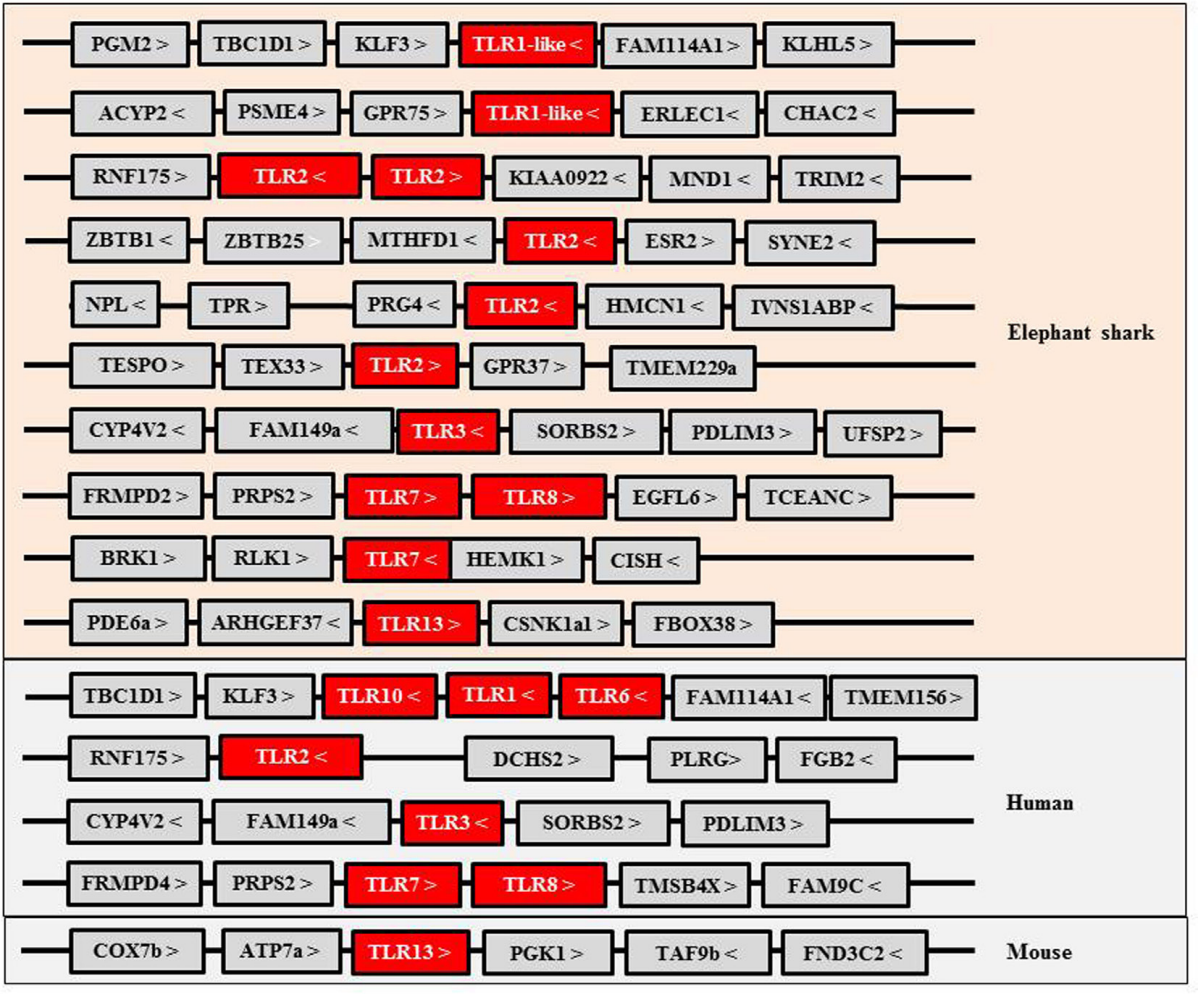
**Gene synteny of Toll-like receptors (TLRs) identified in elephant shark and their homologs in human and mouse**.

Toll-like receptors (TLRs) are an important family of PRRs that activate IFN responses upon activation by intracellular viral/bacterial oligonucleotide pathogen associated molecular patterns (PAMPs). The TLR family consists of 13 members in mammals, and in the elephant shark, some of the oligonucleotide PRRs present have an apparent orthologous relationship with their mammalian counterparts (Figure 1). For example, the gene synteny of two loci harboring the TLR3, 7 (two copies), and 8 genes is conserved between elephant shark and humans. However, curiously, PRRs recognizing bacterial PAMPs, such as LPS (by TLR4) and flagellin (by TLR5), and TLR9 which sense CpG PAMPs, are apparently absent in the current version of the elephant shark genome although present in (at least some) Osteichthyes. A fragmented TLR4 gene is present in the elephant shark genome and is likely a pseudogene, suggesting it had evolved early. In bony fish, TLR4 homologs have been described only in cyprinid species and appear to be unresponsive to LPS (8, 9). Some of the PRRs in the elephant shark, including TLR1-like, TLR2, and TLR7, have duplicated copies, and in the case of TLR2, five gene copies are present. These copies are located in four different loci, one of which is the homologous locus of the human TLR2 gene and contains two tandemly linked TLR2 copies. It seems that TLR6 and TLR10 that flank the TLR1 gene in the human genome likely evolved from one of the TLR1-like genes since only a single TLR gene is present in the homologous locus of elephant shark and bony fish (10–12). Interestingly, both TLR1-like and TLR2 genes have been duplicated in the chicken genome (13). A shark TLR13 is also identifiable, suggesting that TLR13 appeared early in Gnathostome evolution but was retained only in certain vertebrates such as some Osteichthyes and mammals (10, 14, 15) (Figure 1). TLR13 is a member of the TLR11 family, also consisting of fish TLR19–22 and TLR26 (16, 17). These additional fish members of the TLR11 family are not found in sharks, suggesting they may have diverged from the common ancestor with TLR13. It is worth noting that TLR21 exists in birds, amphibians, and bony fish, and avian TLR21 serves as a functional homolog to mammalian TLR9, sensing microbial CpG DNA (13, 16, 18). Some of the TLR family members have been extensively expanded in teleost fish due to the additional whole genome duplications (WGDs), with up to 19 copies identified in some species (16, 18, 19). In addition, the cytosolic sensors activating IFN genes appear to be present in early Gnathostomes.

### Interferons

**Figure 2:**
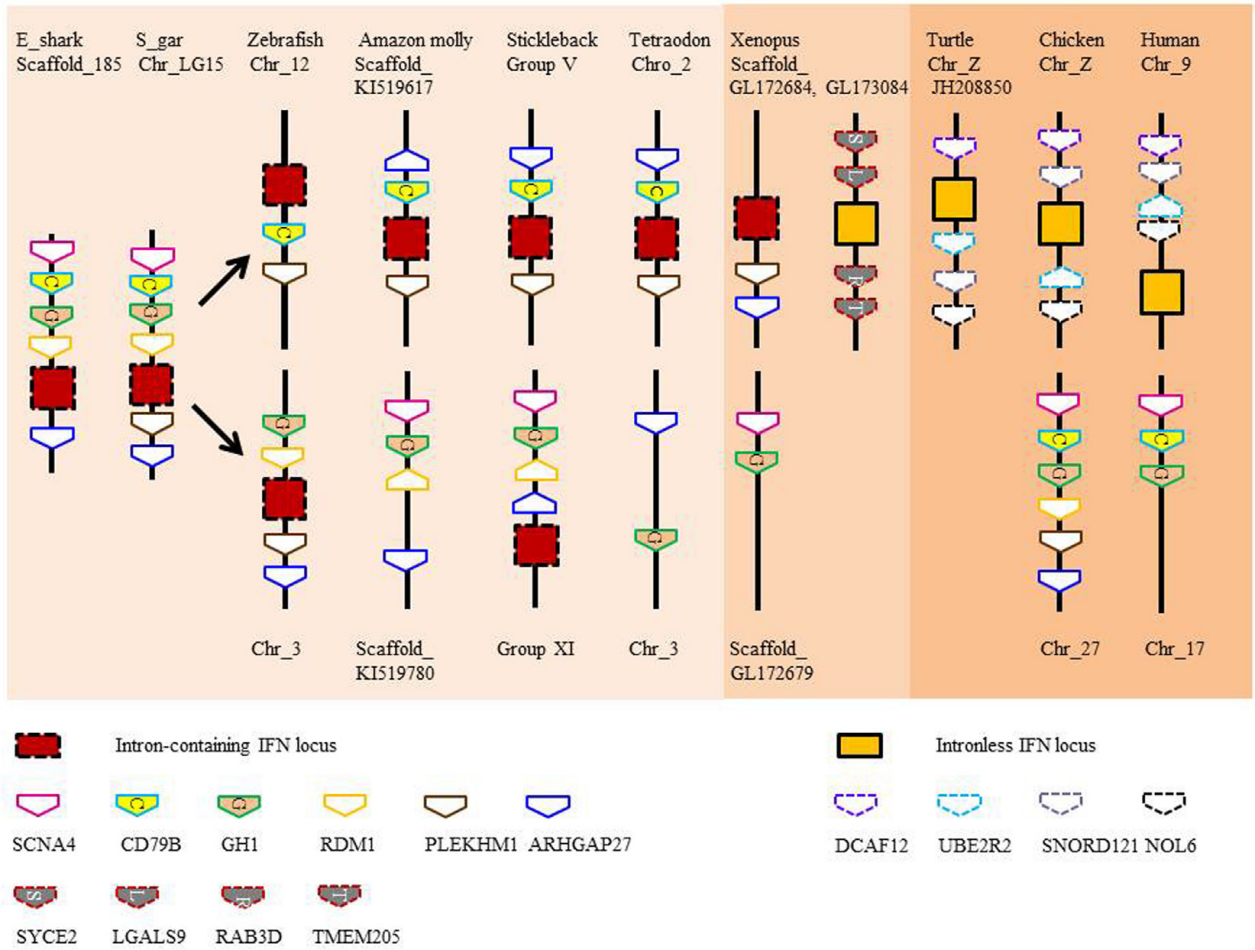
**Gene synteny of type I interferon (IFN) loci in vertebrates**. Note that IFN genes with 2 exons and 1 intron are present in the locus containing intronless genes in scaffold_GL173084 in *Xenopus*.

Type I and II IFN genes, but not type III (also termed IFN-λ), have recently been identified in the elephant shark (3, 20). As in tetrapods, type I IFNs exist as multiple copy genes, while type II IFN is encoded by a single gene. The three type I IFN genes identified in elephant shark are tandemly clustered in the same genomic locus that accommodates the growth hormone and CD79 genes (3), a synteny also seen in bony fish (Figure 2). The single copy type II IFN (IFN-γ) resides next to the interleukin (IL)-22 gene in the elephant shark genome and is located between the MDM1 and DYRK2 gene (3). This chromosomal arrangement has not changed during the evolution of Gnathostomes although additional duplicated homologs can be seen in this locus in some lineages, as with the so-called IFN-γ related (IFN-γrel) gene (see below) and IL-26 gene (21). When modeled against available crystal structures, the shark type I and II IFNs are predicted to comprise multiple α-helices (unpublished data) as is typical of molecules belonging to the IL-10 family (22). Type III IFNs have not been reported in bony fish to date but are present in all tetrapod groups (23–25). This finding hints at a later appearance of these genes during vertebrate evolution or the loss of these genes in the bony fish lineages. However, the quality of the genome sequences and/or a fast divergence rate, the latter known to contribute to the low sequence homology seen in tetrapods, may have hindered their discovery (3, 25).

### Receptors

A complete set of IFN receptors for interaction with IFN ligands are also present in the elephant shark (3). Three putative receptors for type I IFNs, including two copies of IFNAR2/CRFB1–3 and a single copy of IFNAR1/CRFB5, have been reported in the genome cluster also containing the IL-10R2 and IFN-γR2 genes. The expansion of IFNAR2 seems to be common in lower vertebrates, as evidenced in teleosts where up to four copies can be found (26, 27). There are also structural differences of IFNAR1/CRFB5 between fish and tetrapods, notably in the extracellular region where fish IFNAR1/CRFB5 has two rather than four tandem fibronectin-like domains that interact with the IFN ligands and are critical to dictate the actions of individual type I IFNs (4, 7, 27–29). Since the three fibronectin-like domains near the N-terminus (subdomains 1–3) of IFNAR1 are known to be involved in direct binding to the receptors in mammals (30), it will be interesting to determine how fish type I IFN ligands interact with their IFNAR1/CRFB5 receptor. Although the IFN-λ gene has not been found, the existence of its receptor in elephant shark (3) supports the notion that IFN-λ may be present in cartilaginous fish and evolved early.

### Regulation

**Figure 3:**
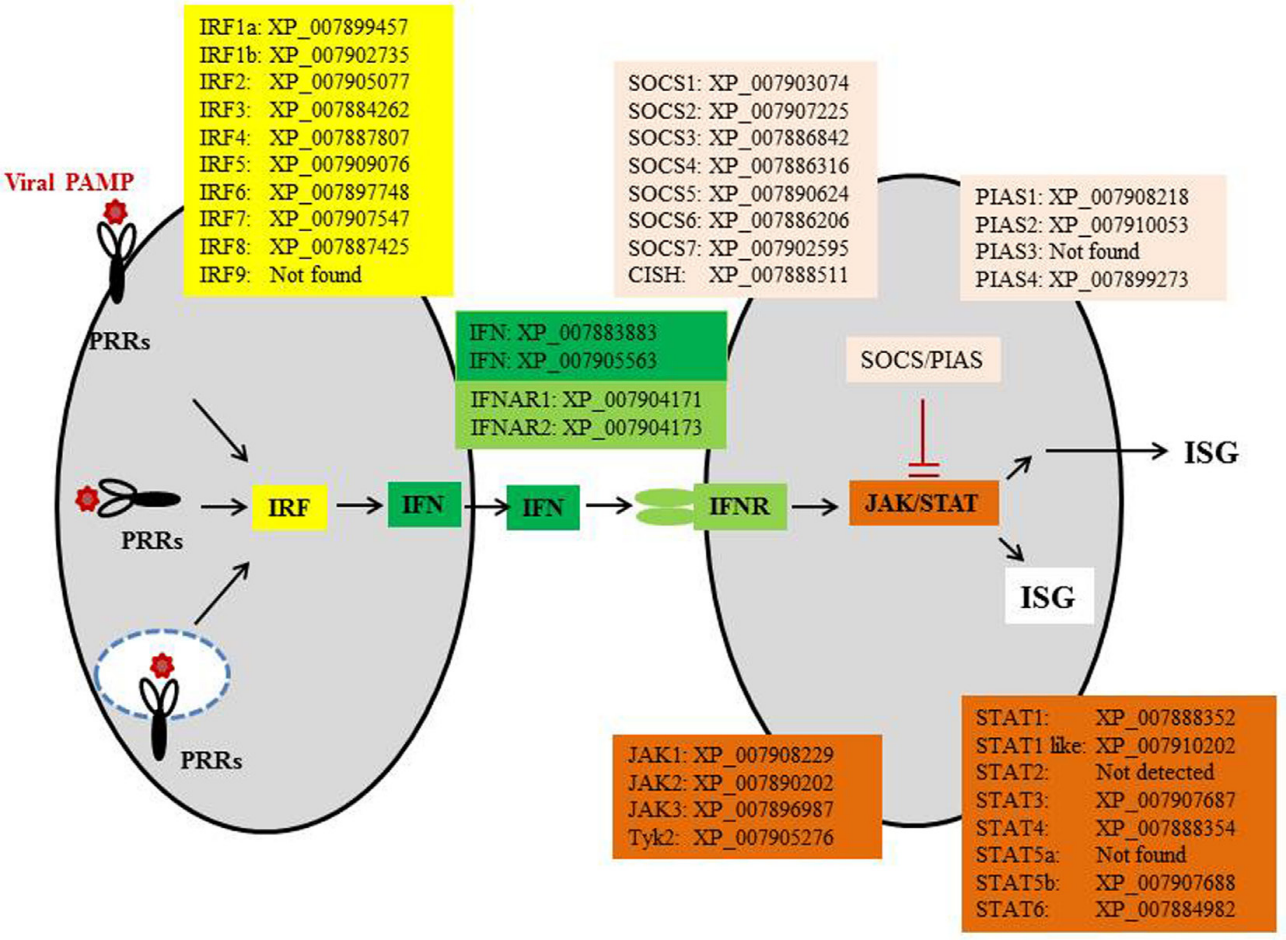
**The IFN pathway of elephant shark**. CISH, cytokine-inducible SH2-containing protein; IFN, interferon; IFNAR, IFN alpha receptor; IRF, IFN regulatory factor; ISGs, IFN-stimulated genes; JAKs, Janus kinases; STAT, signal transducer and activator of transcription protein; PIAS, protein inhibitors of activated STAT; PRR, pattern recognition receptor; SOCS, suppressors of cytokine signaling.

The IFN pathways are coordinated by intracellular signaling molecules. Most of these signaling molecules, including IFN regulatory factors (IRFs), Janus kinases (JAKs), signal transducer and activator of transcription (STAT) proteins, protein inhibitors of activated STAT (PIAS), and suppressors of cytokine signaling (SOCS), are present in cartilaginous fish (Figure 3), as well as in Osteichthyes. Among the IRFs, IRF3 and IRF7 are key regulators for initiation of IFN expression, while IRF4 and IRF8 have opposite roles to inhibit or shutdown the IFN response when viruses are cleared from the host. JAKs, STAT1/2, and IRF9 are essential for IFN signaling, which is in turn negatively regulated by PIAS and SOCS. Homologs of these factors can be traced back to the invertebrates where they have a diverse range of physiological roles in addition to antiviral immunity (31, 32). For example, in fruit fly (*Drosophila melanogaster*), JAK/STAT proteins have been shown to be involved in immune responses to viral and bacterial infections (33, 34). These signaling factors have undergone expansion during the two rounds (2R) of WGDs to provide necessary regulation for the IFN system as it emerged in early jawed vertebrates.

## IFN Gene Structure

**Figure 4:**
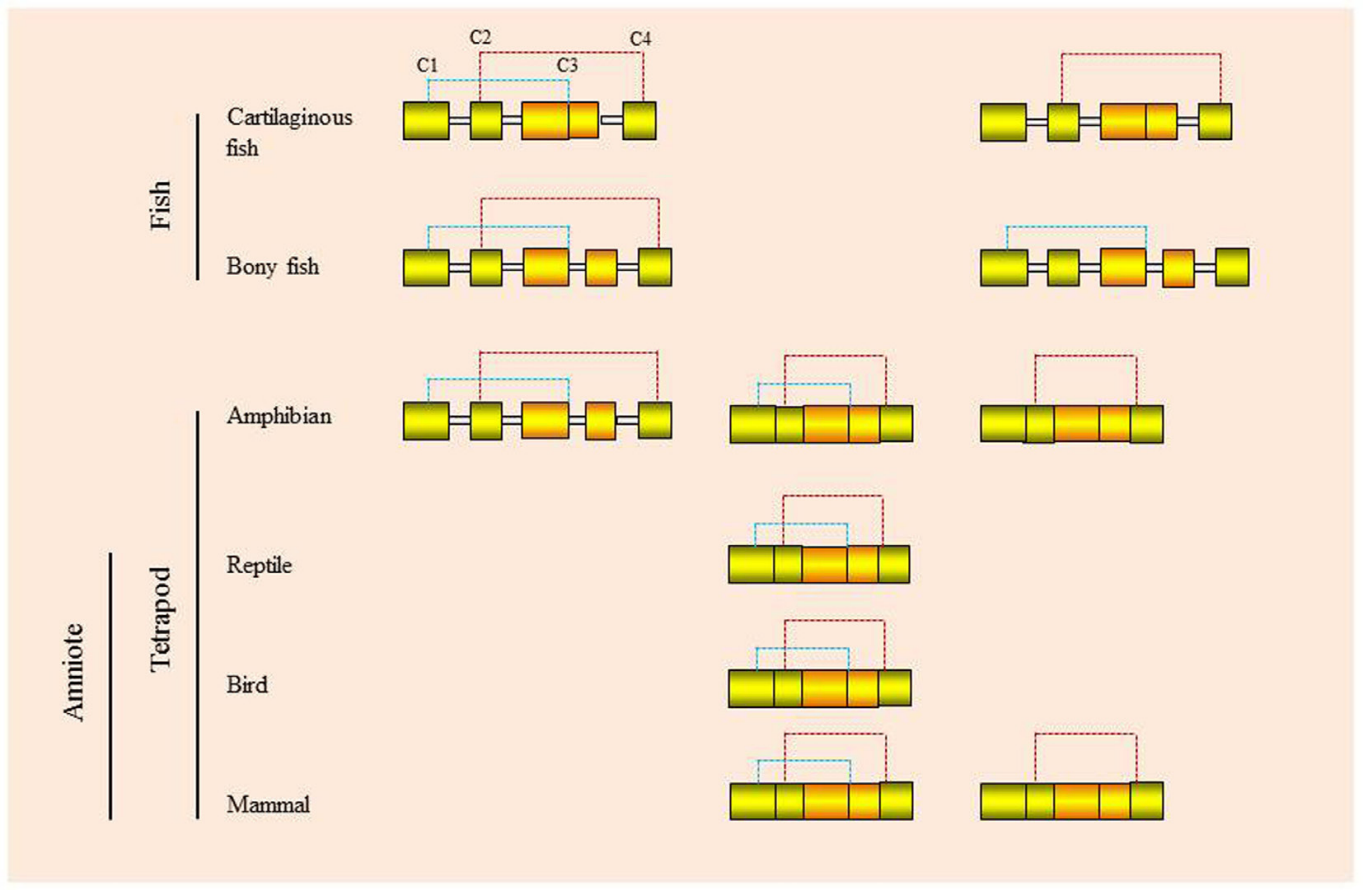
**Genomic organization and putative disulfide bonds of vertebrate type I interferons**.

Molecules within the type II cytokine gene family generally have a 5 exon/4 intron gene organization, as seen in the IL-10 family genes. Within the IFN genes, this gene organization can vary, with examples of intron loss/exon fusion as well as the appearance of intronless genes *via* retrotransposition events (Figure 4). One of the benefits of intronless IFN genes is that they do not require RNA intron splicing for synthesizing functional proteins, hence saving time and energy and eliminating the RNA processing step, which could be targeted by viruses. However, whether this provides a selective advantage still needs to be determined.

**Figure 5:**
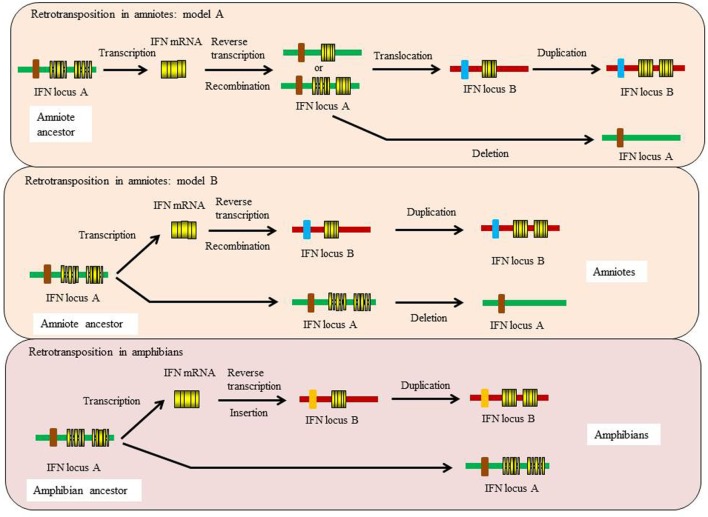
**Proposed models for the independent retrotransposition events of type I IFN genes in amphibians and amniotes**.

This ancestral gene organization of 5 exons/4 introns is seen in type I IFNs in bony fish and in some of the amphibian genes. However, in cartilaginous fish, the IFN genes have a 4 exon/3 intron organization, with apparent loss of intron 3. In amphibians both intron-containing and intronless genes are present (35, 36), while in amniotes (reptiles, birds, and mammals) only the intronless genes are present, with apparent loss of the intron-containing genes. The retrotransposition event that gave rise to the intronless type I IFN genes is thought to have occurred independently in amphibians and amniotes (Figure 5) and highlights the propensity of IFN genes to undergo this phenomenon. Similarly, the type III IFN (IFN-λ) genes have retained the 5 exon/4 intron organization in tetrapods but can also be found as intronless genes in amphibians and mammals, with most being IFN-λ1 variants in the latter case (37). However, many intron-containing type III genes have an additional intron in the upstream region of the start codon. The type III genes have not been found to date in fish, but the presence of the type III receptor genes (IFN-λR1 and IL-10R2) in cartilaginous fish suggests that they exist/existed in this vertebrate group, and also appeared early in vertebrates as predicted from the above model of IFN gene evolution. Lastly, the type II (IFN-γ) genes have a universal 4 exon/3 intron organization from cartilaginous fish to mammals, with loss of the canonical third intron (37).

### The Retrotransposition of Type I and III IFN Genes

Evidence suggests that there were two independent retrotransposition events that led to the appearance of intronless IFN genes in amphibians and amniotes (Figure 5). It is widely believed that one took place in the amniotes, resulting in the insertion of the IFN transcript into the genome locus containing the genes encoding DCAF12 (DB1 and CUL4 associated factor 12), SNORD121A (small nucleolar RNA, C/D box 121A), NOL6 (nucleolar protein 6), and UBE2R2 (ubiquitin conjugating enzyme E2 R2) (Figure 2). However, the identity of the primordial gene whose transcript was involved in the retrotransposition is not clear. Phylogenetic tree analysis suggests that it could have been transcribed from the ancestral gene that gave rise to the IFN1 (accession number: BN001167) or IFN2 (accession number: BN001168) in *Xenopus tropicalis* as they have a much closer relationship with the IFN homologs in amniotes than is seen with other *Xenopus* IFN genes (unpublished data). The IFN transcript utilized in retrotransposition probably had to be expressed in the gonad, making it possible to be integrated into the germline. The appearance of the intronless IFN genes in amniotes may be linked in some way to deletion of the locus containing all the clustered intron-containing IFN genes. The timing of this event is not known but could have occurred simultaneously with the retrotransposition event or sometime later.

Several recent studies demonstrate that a retrotransposition event has also happened in amphibians, independent from the one that occurred in the ancestor of amniotes (35, 36, 38). In both *X. tropicalis* and *X. laevis*, a large number of intronless type I IFN genes have been reported in addition to the intron-containing IFN genes, with 32 (Chr. 03 of *X. tropicalis*) and 26 (Scaffold 20 and Chr. 3L of *X. laevis*) intronless type I IFNs identified in each species (36). In addition, multiple type I IFN genes containing a single intron are present in the intronless IFN gene cluster in both species [(36)—although the present authors were unable to verify this] and potentially reflect retrotransposed genes that have acquired an intron. The mechanisms leading to the remarkable diversification of type I IFNs in amphibians are unclear and are postulated to involve multiple processes including polyploidization, chromosomal duplication, local gene duplication, and retrotransposition (36). These findings are fascinating and highlight the diversity of type I IFN genes in amphibians and the complexity of IFN evolution in the vertebrates. Phylogenetic tree analyses indicate that the intronless IFN genes identified in *X. tropicalis* form a clade with the IFN3–5 molecules (accession numbers: BN001169-711) (35), suggesting that the frog intronless IFN genes may be originated from the transcripts of these genes that seem to differ from the putative ancestral genes (IFN1 and IFN2) giving rise to the intronless genes in amniotes.

The retrotransposition events appear to have had profound impacts on the evolution of type I IFN genes in vertebrates and raises many interesting questions. Such events also make it difficult to establish orthologous relationships when undertaking comparative analyses of functions between intron-containing fish/amphibian IFNs with their amniote counterparts. Intriguingly, the intron-containing and intronless type I IFN genes are regulated in a similar manner, governed by the activation of a panel of conserved PRRs (i.e., TLRs and RIG-I family) and IRFs (i.e., IRF3 and IRF7) (6, 7). For example, the binding sites of IRF3 and IRF7 are present in the promoter regions of both intron-containing and intronless type I IFN genes. How the regulatory mechanisms of type I IFN responses have evolved after the retrotransposition events remains a mystery. Krause proposed a model where the coding exon region (excluding 5′ and 3′ untranslated regions) could be replaced with a double-stranded DNA molecule that is reverse-transcribed from the IFN transcript, leaving the promoter region unchanged (37). It is also possible, as proposed previously, that the DNA recombination could have replaced the entire IFN locus, somehow retaining the promoter region of the most upstream IFN gene, which would contain the necessary regulatory elements (20, 25). Such an event could have taken place prior to the migration of the intronless gene into an alternative locus during genome reshuffling, with subsequent expansion of the intronless genes at this site during evolution (Figure 5).

Retrotransposition has also been detected for the type III IFN genes in amphibians and several mammalian species but not in reptiles and birds (36, 39). This led to the integration of intronless type III IFN genes in the genomes. It has been hypothesized that the retrotransposition events are unrelated and could have occurred independently in various lineages during evolution. An interesting observation is that the intronless type III genes are usually associated with retrotransposons in the genome of both amphibians and mammals (36, 37), which have also been speculated to lead to the remarkable expansion of type I IFN genes in rainbow trout (40). In *X. laevis*, two intronless type III IFN genes are located in a region in Chr. 3L that also contains the intronless type I IFN (36). The two genes are constitutively expressed in kidney, skin, and stomach and can be upregulated in a kidney-derived cell line (A6) by polyI:C and infection with swine influenza virus (TX98 strain), suggesting that they are biologically active in regulating antiviral defense in this species. Similar to amphibians, some mammals possess functional intronless type III genes, but they have not been expanded as much as seen for type I IFN genes.

### Alternative Splicing

The presence of introns allows the potential for alternative splicing, and in some of the teleost type I IFN genes, this can occur at the 5′ end of the transcript (41, 42). This has been shown to generate intracellular forms of the type I IFN molecule that can elicit IFN signaling and induction of ISG expression *via* intracellular IFN receptors (29), as a unique means to combat viral infection. In rainbow trout, the recombinant proteins of the two intracellular forms of type I IFNs generated from a single IFN gene (belonging to the IFN-a subgroup) by alternative splicing have been shown to possess similar functions to the secreted IFN and are able to trigger Mx gene expression in a fibroblast cell line (RTG-2 cells) and protect cells against viral infection. In HEK293 cells with over-expressed intracellular type I IFN and its putative intracellular receptors, induced phosphorylation of STAT1 and STAT2 occurs, suggesting an intracellular IFN system mimicking the actions of secreted type I IFNs exists to be deployed for defending host cells against viral infection (29). Production of intracellular type I IFNs does not require secretion, hence reducing the time and energy for the synthesis in the infected cells, especially at the very early stage of infection, to establish an activated antiviral state. In addition, the intracellular IFN system could provide advantages for the host cells to avoid viral blocking of the IFN secretion pathway and interference of extracellular factors on activation of membrane receptors.

## IFN Gene/Protein Diversity

Multiple genes are commonly present for both type I and type III IFNs. In mammals the large number of type I IFN genes present can be grouped into subtypes, namely α, β, κ, ε, ω/τ, and δ/ζ. Large numbers of type I IFN genes are also present in teleost fish and amphibians, mainly of intronless forms in the latter case. Most of the encoded mammalian IFN proteins have four conserved cysteines (4C), but some possess only two cysteines (2C), as seen with IFN-β and IFN-ε. 4C-containing IFNs are also seen in fish (cartilaginous and bony), amphibians, reptiles, and birds and are thought to represent the ancestral form. Nevertheless, 2C forms of the IFN protein are also seen in cartilaginous and ray-finned bony fish (i.e., not in lobe-finned bony fish—coelacanth) and amphibians, but the pair of cysteines that is retained can differ (Figure 4). Thus, in mammals, amphibians, and cartilaginous fish, it is cysteine 2 and 4 that are retained, while in the 2C subgroups in ray-finned bony fish (holosteans and teleosts), it is cysteines 1 and 3 (20, 28). Interestingly, a recent teleost fish IFN subgroup (termed IFNh) has been described in several perciforme species that groups with the 2C clade but has six cysteines, two of which are aligned in the same position as those in the bony fish group I (2C) type I IFNs (43). Curiously, the perciforme IFNh proteins have an elongated region of approximately 20 aa at the C-terminus and possess similar antiviral functions to the perciforme IFNd previously reported (44, 45). In reptiles and birds only the 4C IFN proteins are known to date, supporting the concept that the 4C form was ancestral and that the 2C forms evolved independently in cartilaginous fish, ray-finned fish, amphibians, and mammals, in the latter two groups following retrotransposition events.

## Impact of WGD in Teleost Fish

The type I IFN locus present in so-called 2R fish (i.e., the gar—a holostean) has both 4C and 2C genes present in a single genomic locus (Figures 2 and 4), and so the ancestor of teleost fish had as a minimal locus one gene of each (4). Hence when the type I IFN locus was duplicated in teleost fish as a consequence of the WGD event that took place at the base of this lineage, two loci were generated as apparent today in species such as zebra fish (28) and stickleback (Figure 2). In zebra fish, it is hypothesized that subsequent gene expansion and loss has meant that one locus now has 2 × 4C genes and one 2C gene, while the second has a single 2C gene (4).

Similarly, while there is a single gene for IFN-γ in most vertebrate groups, in salmonids and cyprinids two genes are present (42, 46) likely due to the WGD events that have occurred independently in these fish lineages. Relatively few comparative studies have been performed of the two paralogs (IFN-γ1 and IFN-γ2), but in general they show near identical tissue expression profiles in healthy fish, as seen in homozygous rainbow trout (42). Similarly, following *in vitro* or *in vivo* stimulation, the expression kinetics are typically similar in trout. Thus, following *in vitro* stimulation with polyI:C (42) or rIL-12 (47) both are upregulated with similar kinetics/doses, as is also seen *in vivo* following DNA vaccination [infectious hematopoietic necrosis virus (IHNV) G protein] or infection with IHNV (42). However, the magnitude of upregulation is often higher for IFN-γ2. In contrast, infection with *Saprolegnia parasitica* or *in vitro* stimulation with rIL-4/13 (a Th2 type cytokine in fish) results in downregulation of both paralogs (15, 48). In cyprinids, the paralogs also have similar expression profiles in healthy tissues, with the exception of gills where IFN-γ1 is dominant (49), and both show antiviral activity when added to GTS9 cells 24 h prior to crucian carp hematopoietic necrosis virus infection (46). They also both have increased expression in scales/epidermis with progression of graft rejection following scale transplantation (50), in kidney cells from allograft-sensitized fish incubated *in vitro* with appropriate allogeneic cells, and following LPS or PHA stimulation of kidney leukocytes *in vitro* (49). Thus, both paralogs of IFN-γ in these species appear to be biologically relevant and have similar regulatory mechanisms.

## IFN-γ Related

While a single type II IFN gene exists in cartilaginous fish, most bony fish (with the exception of salmonids/cyprinids) and amniotes, in some bony fish a second type II gene is present (21, 51, 52). Since it has not been found in gar and coelacanth, it appears to be a teleost-specific tandem duplication. The gene has been termed IFN-γ related (IFN-γrel) (53) since it has relatively low homology to IFN-γ in the same species, and BLAST analysis does not retrieve IFN-γ genes from other vertebrate lineages. However, it does appear to be a type II IFN since it is adjacent to the authentic IFN-γ gene in the genome, has the same gene organization as IFN-γ, and BLAST analysis does retrieve teleost fish IFN-γ molecules. Initial analysis of the sequence revealed that it was truncated at the 3′ end, such that the translated protein apparently lacks a C-terminal nuclear localization signal (NLS) necessary for IFN-γ function, in teleost fish as in other vertebrates (54). However, subsequent studies in ginbuna crucian carp have found that two isoforms of IFN-γrel exist in carps, with the type called IFN-γ-rel1 containing a form of NLS that can translocate GFP into the cell nucleus (i.e., as GFP-KHHHR) (55). His-tagged recombinant IFN-γrel1 protein can also translocate to the nucleus of GTS9 cells after addition to the culture medium, as detected by Western blot analysis of nuclear proteins, unlike the IFN-γrel2 protein and strongly suggests these two types of IFN-γrel signal through different intracellular pathways. Studies of IFN-γrel bioactivity in ginbuna crucian carp have revealed that both forms have antiviral activity and are functional as monomers, in contrast to IFN-γ that is a homodimer (55, 56). *In vitro* studies of IFN-γrel in Rohu (a rel1 with a partial NLS) and in goldfish (IFN-γrel2) have shown it can induce IFN-γR and iNOS expression in cultured leukocytes, with additional effects seen on IL-1β, TNF-α, IL-8, and ceruloplasmin expression in goldfish cells (52, 57). Stimulation of tetraodon spleen and head kidney cells *in vitro* with IFN-γrel2 (termed IFN-γ1 in this paper) enhanced their nitric oxide responses and expression of ISG15 (58). Injection of IFN-γrel2 into Japanese pufferfish has been shown to increase phagocyte function in terms of phagocytosis and ROS production, and IFN-γ expression in head kidney cells 1 day post-injection, with a longer term effect seen on IL-6 and IL-12p35/IL-12p40 gene expression (59). It is clear that IFN-γrel is an important immune molecule within the immune system of teleost fish.

## IFN Receptors

Six receptor molecules are known to interact with type I, II, and III IFNs in mammals. Although existing as multiple isoforms, type I IFNs bind to the same protein complex consisting of two subunits of the receptor chains IFNAR1 and IFNAR2. Similarly, the type II IFN (IFN-γ) signals through a receptor composed of IFN-γR1 and IFN-γR2, and all the type III IFNs share the same receptor complex of IFN-λR1 (IL-28R1) and IL-10R2. The genes encoding these receptors are found in three genome loci where synteny of these genes has rarely changed during Gnathostome evolution (26, 27). The IFNAR1, IFNAR2, IFN-γR2, and IL-10R2 genes are clustered in a single region except in zebrafish where the IFN-γR2 gene is located in Chr. 9, while the genes encoding IFNAR1, IFNAR2, and IL-10R2 are in Chr. 5 (27). The IFN-γR1 gene is linked with IL-20Ra and IL-22Ra2, which are flanked by the genes encoding Olig3 and SLC35d3 (26, 60, 61). Lastly, the IFN-λR1 gene found in elephant shark and tetrapods resides next to the IL-22Ra1 gene in the genome. It has not been identified in bony fish where the IFN-λ gene is thought to have been lost.

While few receptor-binding studies have been performed out with the mammals, interestingly, in teleost fish type I IFNs bind to distinct receptors in stark contrast to the findings in mammals. While a single IFNAR1 is present in most species (such as zebra fish and tetraodon), multiple forms of IFNAR2 exist, generated by local gene duplications. In zebrafish, it has been shown that the two IFNAR2s (CRFB1 and CRFB2) preferentially bind to group I (containing two cysteines) and II (containing four cysteines) type I IFNs, respectively (28). In Atlantic salmon, interaction of type I IFNs with the receptors is even more complex since both IFNAR1 and IFNAR2 have been multiplied, with four copies of each identified at two different chromosomes (Chr. 21 and 25); namely salmon CRFB5a, 5b, 5c, and x are homologs of IFNAR1, while CRFB1a, 1b, 2, and 3 are homologs of IFNAR2. It has been speculated that the increased copies of IFNAR1 and IFNAR2 are due to the salmonid-specific WGD. Binding to the different IFNAR1 isoforms by the IFN subgroups is possible and may allow differential cellular signaling. For example, salmon IFN-c binds CRFB5a or CRFB5c, while IFN-b may signal through a receptor with CRFB5x (27).

In addition to the above differences in gene number and ligand binding, the protein structure of fish and tetrapod IFNAR1 displays a striking difference. Fish IFNAR1 homologs have only two predicted fibronectin domains in the extracellular region, while tetrapod IFNAR1 possess four fibronectin-like domains, possibly due to a domain duplication that occurred in the tetrapod ancestor. It is worth noting that the structural change of the receptor likely took place before amphibians diverged from the main vertebrate lineage, preceding the IFN retrotransposition events (including those in amphibians). In mammals, all four fibronectin domains are shown to be involved in receptor binding. With only two such domains, how fish type I IFNs form a complex with the receptors is a mystery, especially as crystal structural analyses indicate that fish type I IFNs are structurally similar to that of their mammalian homologs, consisting of six α-helices (22).

As with type I IFNs, in teleost fish, the two members of the type II IFN family that are present (IFN-γ and IFN-γrel) appear to interact with different receptors. In zebrafish, which have a single copy of IFN-γR2/CRFB6, both IFN-γ and IFN-γrel have been shown to induce expression of downstream genes through CRFB13 and CRFB17, respectively (61, 62). However, a recent study demonstrates that tetraodon IFN-γ binds equally to both CRFB13 and CRFB17 expressed in transfected COS cells (63), with weaker binding of IFN-γrel to CRFB13 than to CRFB17. Some cyprinid and salmonid species possess duplicated copies of IFN-γ, IFN-γrel, and the receptor chains, making determination of the pairing relationships between ligands and receptors complicated. For example, two copies of IFN-γR2/CRFB6 as well as IFN-γ and IFN-γrel have been described in Atlantic salmon, rainbow trout, and ginbuna crucian carp (27, 42, 46). In ginbuna crucian carp, it has been shown that the two IFN-γ paralogs exhibit specific binding to different receptors (46). Interestingly, elephant shark also has two copies of the IFN-γR1 gene, which are tandemly arranged in the genome, one of which has a short intracellular region containing well-conserved binding motifs for JAK1 and STAT1. Whether these IFN-γ receptors are functional remains to be investigated.

## Conclusion

We have learnt a lot about IFN and IFN receptor genes throughout the jawed vertebrate classes, in large part due to the sequencing of the genome of increasing numbers of species. While functional studies lag behind in many cases, studies in fish (especially teleosts) have demonstrated their important role in antiviral defense in early vertebrates as seen in mammals. It is clear that IFN genes have undergone extensive expansion in many lineages, in some cases associated with the generation of intronless genes following retrotransposition, and in other cases following WGD events. The protein cysteine pattern appears to define IFN types in most vertebrate classes, with loss of cysteine 1 and 3 having apparently occurred independently in cartilaginous fish, amphibians, and mammals. The loss of cysteines 2 and 4 in ray-finned fish appears unique and demonstrates the plasticity of the IFN molecule. It is likely a few surprises regarding IFN gene function in different vertebrate groups are still to be uncovered.

## Author Contributions

Both authors have made substantial, direct, and intellectual contribution to the work and approved it for publication.

## Conflict of Interest Statement

The authors declare that the research was conducted in the absence of any commercial or financial relationships that could be construed as a potential conflict of interest.
